# Prenatal care counseling and delivery method among women with multiple Cesareans: A cross-sectional study from Democratic Republic of Congo

**DOI:** 10.1371/journal.pone.0238985

**Published:** 2020-11-09

**Authors:** Raha Maroyi, Nyakio Ngeleza, Laura Keyser, Katenga Bosunga, Denis Mukwege

**Affiliations:** 1 Panzi General Referral Hospital, Bukavu, The Democratic Republic of Congo; 2 Université Evangélique en Afrique (UEA), Bukavu, The Democratic Republic of Congo; 3 Mama LLC, Boston, MA, United States of America; 4 Université de Kisangani, Kisangani, The Democratic Republic of Congo; Lausanne University Hospital: Centre Hospitalier Universitaire Vaudois (CH), SWITZERLAND

## Abstract

Prenatal care (PNC) and counseling about delivery method is an important strategy to prevent delivery complications among women with multiple prior Cesarean sections (CS). In low income countries, an elective CS is recommended for this population. This cross-sectional study examined factors associated with counseling about delivery method and its influence on the likelihood of an elective CS delivery. A total of 422 women with ≥2 prior CS who delivered across five hospitals in Democratic Republic of Congo (DRC) were interviewed about PNC and counseling. Descriptive statistics and multivariate regression were completed to ascertain factors associated with counseling. Only 33.6% delivered via planned CS; 60.7% required an emergency CS. One-quarter completed four PNC visits; 64.5% received counseling. Number of PNC visits and number of prior CS were significant predictors of receipt of counseling. Women who received ≥2 PNC visits were 2.2 times more likely to have received counseling (p = 0.000). Among women who received counseling, 38.6% had a planned CS compared with 24.7% in the non-counseled group. Counseling was associated with mode of delivery; emergency CS and vaginal delivery were more frequent among women who did not receive counseling (p = 0.008). These findings highlight the importance of counseling during PNC visits. This study also highlights the poor coverage and quality of counseling in this high-risk population and the need for improvements in PNC. Less than 40% of counseled women followed provider recommendations for a planned delivery via CS. The majority labored at home and later delivered emergently. The significant number of women who trial labor without medical supervision despite their high-risk status sheds light on the influence of patient perceptions about CS and acceptance of medical intervention during birth.

## Introduction

Access to Cesarean section (CS) is an important component of obstetric care, particularly in an emergency context, and is associated with reduction in maternal and neonatal mortality and morbidity [1]. A CS rate of 5–15% of births is considered optimal to achieve gains in maternal and child health indicators. However, this percentage remains low in many low- and middle-income countries (LMICs) and is disparate according to sociodemographic factors within countries, reflecting barriers to access, such as lack of health facilities in rural areas and low population health literacy [2]. While efforts to improve obstetric care, including prenatal care (PNC) and access to CS have led to improvements in facility-based births, much work remains to ensure women in LMICs receive adequate PNC, including counseling about labor and delivery. In fact, a recent review reports on CS-associated maternal deaths in LMICs, citing a high mortality rate that remains unchanged over time and linking emergent CS and those performed during second stage labor with significantly increased mortality and morbidity [3].

Among LMICs, a history of prior CS is often cited as the indication for a repeat CS [3]. While research supports a trial of labor after CS, the majority of this literature draws from high income countries (HICs), where pregnancy monitoring is accessible, and fertility rates are relatively low [4]. In LMICs this remains a source of debate with conflicting research on complications associated with a trial of labor versus planned CS [5, 6]. One publication from the Royal College of Obstetricians and Gynecologists highlights the risks associated with a trial of labor or vaginal birth after CS (VBAC) specifically in the context of Sub-Saharan Africa [7]. The authors cite complications of VBAC in this setting, including uterine rupture, hysterectomy, venous thromboembolism, hemorrhage, organ damage, and death. Availability of blood transfusions is incredibly limited in this region. Fetal monitoring via ultrasound is also quite sparse, thus limiting detection of potential complications during PNC visits, such as placental abnormalities [7]. In the absence of adequate obstetric monitoring and care provision, it remains a recommendation for elective repeat CS, particularly in the context of a history of multiple CS.

In any circumstance, indications for a trial of labor after CS require adequate supervision and readily available emergency CS. Because this may be difficult to achieve in LMICs, regional practices and policies often advocate for a planned CS for women with prior CS. These practices, coupled with high fertility rates in many LMICs, raise concern for women with multiple prior CS.

In the Democratic Republic of Congo (DRC), the fertility rate is 6 births per woman [8], and the country’s CS rate is 5%, though approaches 10% in the eastern province of South Kivu [9]. In DRC high rates of maternal and perinatal mortality are associated with CS conducted in an emergency context. For these reasons, national guidelines support planned CS for women with history of multiple CS [9]. Accordingly, PNC for women with multiple prior CS is imperative. In DRC it is common for women to obtain PNC from various cadres of providers at local health facilities, including nurses, midwives, and community health workers. PNC visits typically include patient registration and medical history, assessment of vital signs and weight, health and nutrition education, HIV testing and counseling, and obstetric examination. Though ancillary staff are skilled in these standard components of PNC, patient evaluation and education about recommended mode of delivery is often deferred to the obstetrician. Unfortunately, women do not always complete the recommended minimum of four PNC visits [10]. Additionally, due to a limited number of physicians per capita and a high fertility rate in DRC, women may not consult an obstetrician prior to delivery.

The present study documented the delivery patterns in women with multiple prior CS in South Kivu, DRC, in order to understand whether attendance at PNC and specifically, counseling about mode of delivery during PNC visits was associated with planned CS. Factors associated with receipt of counseling about mode of delivery were also examined. Findings from this research may inform local, national and regional efforts to improve maternal and perinatal health outcomes and contribute to our understanding of barriers and challenges associated with PNC in LMICs.

## Materials and methods

### Study design

This is a cross-sectional study conducted from September 2015 through May 2016 in the maternity wards of five tertiary care hospitals in the South Kivu, DRC. These are general reference hospitals with a technical platform capable of providing emergency obstetric care (EmOC). All thirty-two general reference hospitals in South Kivu province were stratified into three categories: rural, suburban and urban. Sites were then randomly selected to include two rural and two suburban hospitals. Only one urban hospital functions in this region and was included in this study.

### Study population

The eligible study population included all women delivering at one of the selected hospitals with a history of 2 or more prior CS. Women were included if they delivered vaginally or via planned or emergency CS. Women were excluded if they presented with other causes of gynecological scarring or if they had history of one or no CS. The study was approved by the Institutional Ethics Committee (CIE) of the Catholic University of Bukavu (UCB) (UCB / CIE / NC / 010/2016).

### Sample size

It was determined that 384 women were required to achieve 80% power with an alpha of 0.05. To account for potential item non-response, this was increased by 10% for a target sample size of 422 women. The proportion of women recruited in each hospital was proportional to the number of births delivered in that hospital in the prior year (Table 1).

**Table 1:** Sample size distribution by hospital.

Hospital	Type	Total Number of Cesarean Deliveries in 2014	Number of Women Recruited During Study Period (% Total)
Ifendula	Rural	604	79 (19)
Kalonge	Rural	394	52 (12)
Nyantende	Suburban	398	52 (12)
Rau	Suburban	668	88 (21)
Panzi	Urban	1147	151 (36)
TOTAL		3211	422 (100)

### Data collection

Eligible patients were screened on registration at the hospital and recruited on arrival in the obstetrics department. If women required EmOC, they were screened and recruited within twenty-four hours after delivery. After obtaining written informed consent, medical staff conducted patient interviews and completed a study-specific questionnaire, including demographics, patient characteristics and mode of delivery (S1). All women who consented were interviewed after their delivery. Enrollment continued during the study period until the target sample size was reached at each hospital.

During a 15-minute structured interview, women were asked to report on sociodemographic characteristics, obstetric history, and PNC during the current pregnancy. Each woman was asked whether or not she had attended PNC (yes/no), and if so, the number of visits (0, 1, 2, 3, 4+). For women who reported attending at least one PNC visit, they were also asked (1) whether they received counseling about the recommended delivery method (yes/no), and if yes, (2) what mode of delivery was recommended (planned CS or vaginal delivery). Mode of delivery was abstracted from the clinical record and classified as a planned CS, emergency CS with trial of labor, or vaginal delivery.

### Statistical analyses

Data were analyzed using SPSS version 20 (IBM, Armonk, NY, USA). Frequency distributions, means and standard deviation were examined. Cross-tabulations of each variable were examined, and Chi-square tests were calculated as appropriate to assess factors associated with PNC visits, PNC counseling, and mode of delivery. Multivariate regression analysis was conducted to assess patient characteristics associated with receiving PNC counseling. Significance was defined as p <0.05.

## Results

In this cross-sectional sample, 422 women with multiple prior CS were interviewed. Demographic information and patient characteristics are reported in Table 2. The average age at delivery was 28.8 years (range 18–45 years). Nearly one-third of women reported no education (31.5%), while 19.2% completed primary school and 36.7% completed secondary school. With regard to obstetric history, 52.1% reported having had 2 prior CS, 30.1% reported 3 prior CS, and 17.8% reported 4 or more prior CS. Approximately three-quarters of women attended at least 2 PNC visits; 25.6% completed four or more visits, while 14.0% received no PNC (Table 2). Among women who received any PNC, 64.5% reported they received counseling regarding recommended mode of delivery. Emergency CS following a trial of labor was the dominant mode of delivery (60.7%). One-third (33.6%) delivered via scheduled CS, and 5.7% delivered vaginally. The indications for emergency CS following a trial of labor are summarized in Table 2. These included: contracted pelvis (55.1%), hemorrhage (6.6%), uterine rupture (12.1%), abnormal fetal presentation (7.0%), and fetal distress (19.1%).

**Table 2:** Demographics and patient characteristics (N = 422).

Age	Total, n	Total, %
<25	87	20.1
25–29	154	36.5
30–34	116	27.5
>/ = 35	65	15.4
**Education**		
None	133	31.5
Primary	87	20.6
Secondary	155	36.7
University	18	4.3
Unknown	29	6.9
**Parity**		
2	11	2.6
3	129	30.6
4+	282	66.8
**Number of Previous CS**		
2	224	53.1
3	123	29.1
4+	75	17.8
**Number of PNC Visits Attended**		
0	59	14
1	39	9.2
2	113	26.8
3	103	24.4
4+	108	25.6
**PNC Counseling**		
Yes	272	64.5
No	150	35.5
**Mode of Delivery**		
Vaginal	24	5.7
Planned CS	142	33.6
Emergency CS with Trial of Labor	256	60.7
**Indications for Emergency CS (n = 256)**		
Contracted pelvis	141	55.1
Hemorrhage	17	6.6
Uterine rupture	31	12.1
Abnormal fetal presentation	18	7.0
Fetal distress	49	19.1

A variety of factors were associated with receipt of PNC counseling about delivery method, and these are highlighted in Table 3. The number of prior CS (p = 0.013), the number of PNC visits (p<0.000) attended, and mode of delivery (p = 0.008) were significantly associated with PNC counseling, while age and education were not related.

**Table 3:** Association of PNC counseling with patient characteristics & mode of delivery.

Variable	PNC Counseling Received, n (%)		Chi-square	p-value
	Yes (N = 272)	No (N = 150)		
**Age**				
<25	60 (22.1)	27 (18.0)	1.469	0.68
25–29	100 (36.8)	54 (36.0)		
30–34	73 (26.8)	43 (28.7)		
>/ = 35	39 (14.3)	26 (17.3)		
**Education**				
None	80 (29.4)	53 (35.3)	1.98	0.15
Primary	57 (20.9)	30 (20.0)		
Secondary	102 (37.5)	53 (35.4)		
University	16 (5.9)	2 (1.4)		
Unknown	17 (6.3)	12 (8.0)		
**Number of Previous CS**				
2	132 (48.5)	88 (58.7)	2.47	0.013
3	83 (30.5)	44 (29.3)		
4+	57 (21.0)	18 (12.0)		
**Number of PNC Visits Attended**				
0	0 (0.0)	59 (39.3)	131.27	<0.000
1	26 (9.5)	13 (10.5)		
2	84 (30.8)	29 (19.3)		
3	71 (26.1)	32 (21.0)		
4+	91 (33.4)	17 (11.3)		
**Mode of Delivery**				
Vaginal	12 (4.4)	12 (8.0)	9.48	0.008
Emergency CS with Trial of Labor	155 (57.0)	101 (67.3)		
Planned CS	105 (38.6)	37 (24.7)		

Results of multivariable regression analysis are illustrated in Table 4. The number of PNC visits was dichotomized, 0–1 versus 2+ visits. Women who received 2+ PNC visits were 2.2 times more likely to have also received counseling specific to delivery method during those visits (p = 0.000). The likelihood of counseling about delivery method also increased with number of prior CS (OR 1.70; 95% CI 1.24–2.31).

**Table 4:** Factors associated with receipt of PNC counseling about delivery method.

Variables	Odds Ratio (Ajusted)	Confidence Interval, 95%		Z-Statistic	P-Value
		Min	Max		
**Age**	0.9545	0.9121	0.9988	-2.0102	0.0444
**Education**	11.2438	1.1527	109.6743	2.0822	0.0373
**Number of PNC Visits** (0–1 versus 2+ visits)	2.2192	1.8363	2.6819	8.25	**0.0000**
**Number of prior CS**	1.7003	1.2496	2.3135	3.3781	**0.0007**

Among women who received counseling about delivery method, 38.6% had a planned CS compared with 24.7% in the non-counseled group. Counseling was associated with mode of delivery: emergency CS and vaginal delivery were more frequent among women who did not receive counseling.

## Discussion

This study is the first clinical evaluation of the association of PNC and counseling about delivery mode with having a planned CS among women with multiple prior CS in eastern DRC—a region characterized by high rates of fertility, maternal mortality and obstructed labor. Nearly two-thirds of women in this study required EmOC, with only one-third having a scheduled delivery by non-emergency CS. Women who attended two or more PNC visits were more likely to receive counseling about mode of delivery, which did increase the likelihood of a planned CS. It is concerning that one in seven women did not attend any PNC visits, despite their high-risk status, and a significant proportion of women who did attend PNC were not counseled about the recommended mode of delivery. Our findings highlight the need for health policies and programs to more effectively target this group of women with high-risk pregnancies.

Only one-quarter of the women had completed the minimum recommended four PNC visits. Our data are consistent with national data from the Ministry of Health that has reported that 12% of pregnant Congolese women receive no PNC [9]. This further supports the need to address barriers to PNC, including transportation, cost, and general lack of health services coverage. Similarly, research from Mali [11] and Togo [12] concluded that inadequate PNC contributed to increased rates of emergency CS rates and associated poor maternal and perinatal outcomes.

Importantly, only 64% of women in the study received counseling, and of those, only 38.6% delivered via planned CS. While counseling did increase the likelihood of a planned delivery, the majority of women still delivered via emergency CS. This finding requires further study to understand the reasons that women continue to risk a trial of labor, despite medical recommendations. Previous studies in Africa have examined patients’ perceptions about CS, finding that many women are averse to the procedure, citing fear, guilt, and shame associated with a surgical birth [13–16]. Religious beliefs and sociocultural norms influence these negative views about CS [13–16], and in one study, 22% of women refused the procedure [15].While similar studies have not been conducted in DRC, anecdotally, health care providers commonly hear similar beliefs among women, and negative attitudes are often perpetuated by local religious and traditional leaders. This may explain the low uptake of planned CS, even when women receive counseling about delivery method, and supports the need for urgent action to address population health literacy, to improve trust in the health system, and to dispel negative traditional and cultural beliefs about CS.

Both PNC attendance and counseling about delivery method must be augmented to ensure that women understand the maternal and perinatal risks of attempting a vaginal delivery, particularly in a context with inadequate supervision. In light of recent research that supports a trial of labor among women with one or two prior CS in LMICs [6], it is possible that national and regional guidelines may shift. However, findings from our study highlight significant challenges with low completion of at least 4 PNC visits, limited counseling provided to women, and even in the context of counseling, low compliance with provider recommendations.

Shortcomings in the number, timing and content of PNC could be improved by changing the follow-up approach for high-risk pregnancies after multiple CS and incorporating consultation with an obstetrician or general practitioner in women’s third trimester. Additionally, inclusion of a midwife during PNC counseling would likely provide reassurance about elective delivery. Discussions regarding the choice of hospital and the process for planning a delivery could take place with potential barriers to hospital access being addressed during this consultation. Encouraging women to plan to deliver at a tertiary care facility is also important to ensure adequate resources are available for complex deliveries. Given the low proportion of women completing the four PNC visits, specific targeting of this high-risk group is needed, with consideration given to the modest resources available, infrastructure difficulties, and geographical barriers in LMICs.

This cross-sectional study has some limitations. The sample includes hospital-based deliveries only, and so may not be representative of all women, a proportion of whom deliver at home or in local facilities. However, we did obtain a representative sample of hospital births, and 84% of births in South Kivu take place in a health facility [17]. We did not have information regarding women’ reasons for undergoing a trial of labor or on barriers to obstetric care, thus we could not assess the impact of counseling on delivery method independent of these other factors. Additionally, the details of PNC counseling remain unknown. The type of provider (midwife, physician, community health worker, etc.) and aspects of the discussion, including patient impression and stated concerns may influence adherence to recommendations.

Research is needed to increase understanding of the concerns of women with a history of multiple CS, as well as to identify the specific barriers they face in accessing PNC. Evaluation of obstetric practices and decision-making protocols that lead to CS may be warranted to ensure adherence to guidelines, particularly for a woman’s first CS. Improved adherence to these guidelines may reduce the need for multiple CS. Additionally, as a small proportion of women in this study had a successful vaginal delivery, information on their obstetric profile would help guide recommendations regarding options for vaginal birth after CS in LMICs.

## Conclusions

In DRC, a significant number of women with multiple prior CS delivery via emergency CS. For this high-risk group, attempting a trial of labor, particularly outside a hospital setting, constitutes a significant obstetric risk. PNC and counseling about delivery method improved the likelihood of a planned CS; however, coverage and quality of these visits remains inadequate. To avoid delivery complications, increased focus on delivery planning and an assessment of risks of vaginal birth during PNC is much needed. Including at least one PNC consultation with a general physician or obstetrician for this high-risk population may improve maternal and perinatal outcomes. Future research should identify women’s concerns about and barriers to planned CS. Community sensitization and transfer of women for third trimester medical consultation may improve women’s acceptance and adherence to a delivery plan.

## Supporting information

S1 File (PDF)

S2 File (PDF)

S1 Database (XLSX)
