# Supplementary material for: Prenatal care counseling and delivery method among women with multiple Cesareans: A cross-sectional study from Democratic Republic of Congo
Source: PLoS One. 2020 Nov 9;15(11):e0238985. doi: 10.1371/journal.pone.0238985 (PMC7652330; doi:10.1371/journal.pone.0238985)
Supplement: S1 File — (PDF) [file pone.0238985.s001.pdf]

MULTIPLE CAESAREAN SECTION (2 AND MORE) QUESTIONNAIRE

Site ___________________________________Code n ° ________________

I. IDENTITY

- Age (Years): __________ Profession__________ Address:
- -Home-Hospital distance: <50 ☐ Km 50-150 Km ☐ > 150 Km ☐
- Education Level: ______________
- Mode of Admission: **refered Yes / No**
- If yes , From : ☐ Hospital ☐ Health ☐ Center Clinic

☐ ☐ • traditionalpractitioner

Prayer Room

II. OBSTETRIC HISTORY

- Obstetric Formula : G P A L
- Age of current Pregnancy (weeks) : ___ Prenatal care consultation Yes / No Number :__
- Birth interval space :

  <18 months ☐

  18-24 months ☐

  > 24 months ☐

- Number of previous CS : ___
- Indications for previous cesareans: **1** **st**_________ **2** **nd** _________**3** **rd** _________4th_________5th_________6th_______________

- **Counseling on *current delivery method* done ? Yes / No**

If yes what method : Caesarean section ☐

vaginal ☐

III. EXAMINATION INFROMATIONS

- Date of Arrival at the Hospital:_____________________Time____________

- Blood pression (mm Hg): __Breathing rate___ Heart rate(beats) __ **T °** __

- Hb / number ________ g / l Blood group made: YES/NO Ultrasound YES/NO Platelets YES/NO HIV serology YES/NO
- Fetal heart rate : Normal / pathological / Absent

- Parturient come in labor: YES/ NO

- Open partogram YES/ NO
- TALAC YES / NO
- Vaginal delivery YES / NO
- Elective Caesarean delivery YES / NO
- Caesarean delivery during labor: YES/ NO

  1. emergency caesarean
  2. Extreme Emergency Caesarean section
  3. indication_________________

IV. PROCEDURE

- **Date of** delivery and / or caesarean decision ______________ Time_________
- **Start time of caesarean section ______End time_________**
- Time **fetal extraction** __________________
- Surgeon: ___________________________Type of anesthesia __________
- Type of Skin incision ___________________Antibioprophylaxis

Done YES/NO

- Caesarean section + other added procedures: No ☐ Yes ☐
  - Hysterectomy ☐
  - Bilateral Tubal ligation ☐
- Operating reports:
  - o **Dense adhesions**
  - o **Uterine rupture**
  - o **Apoplexy (Couvelaire Syndrom)**
  - o **Uterine atony**
  - o **Placental anomalies (acreta, percreta, increta)**
  - o **Placenta Prævia**
  - o **Intestinal injury**
  - o **Bladder injuriy**
  - o **Orthers (specify)___________________**

V. MATERNAL PROGNOSIS

Forecast: Good / Bad

Transfusion: **No** ☐

Yes ☐ Number of blood units : 1/ 2/ 3/ 4/ >4

VI. FETAL PROGNOSIS

o **APGAR at the fifth minute : 0** ☐ **1-4** ☐ **5-7** ☐ **8-10** ☐

o Resuscitation of the Newborn: **Yes / No**

o **Weight _________grs Gender: M/F**

o Macerated death YES/ No

o Respiratory Distress: **Yes**

Demise fetus Yes/ No

Neonatal mortality Yes / No

VII. Maternal POST OPERATING outcome

- Reintervention: **Yes / No**
- **Complications after childbirth** (Low and Cesarean section): (CHECK IF PRESENT)
  o *Blood loss :*
    - *over 1500ml*
    - *between 1000- 1500 ml*
    - *less 1000 ml*

  o *Internal bleeding*
  o *surgical site infection*
  o *Eventration, Evisceration*
  o *Foreign body*
  o *Anesthetic complications*
  o *Pelvi peritonitis*

  o *acute pulmonary oedema*
  o *Pyelonephritis*
  o *Endometritis*
  o *Post-operative intestinal occlusion,*
  o *Paralytic* intestinal *ileus*
  o *venous thromboembolism (VTE)*

- maternal Death: **Yes /No**

VIII. AT DISCHARGE

- **Day of hospital Discharge :**

Before 7D ☐

On the 7th day ☐

Between 7-14 J ☐

14 to 21 J ☐

Beyond 21 days ☐

- contraception offered:

Yes ☐ Suggested method: ________________

No ☐
