# Supplementary material for: Prenatal care counseling and delivery method among women with multiple Cesareans: A cross-sectional study from Democratic Republic of Congo
Source: PLoS One. 2020 Nov 9;15(11):e0238985. doi: 10.1371/journal.pone.0238985 (PMC7652330; doi:10.1371/journal.pone.0238985)
Supplement: S2 File — (PDF) [file pone.0238985.s002.pdf]

ELEMENTS DU PROTOCOLE SUR LA CESARIENNE (2 ET PLUS)

Site ____ Code n°_________________

I.IDENTITE Nom de la Patiente :

- Age(Ans) :___________________________Profession______________________________
- Adresse : -Lieu___________________________________

  -Distance Maison-Hôpital : ☐ **< 5O Km** ☐ **50-150 Km** ☐ **> 150 Km**
- Niveau d'instruction : _______________________
- Mode d'Admission : **1) Seule** ☐

  2) Référée ☐ : de l'Hôpital ☐ C .S ☐ CH ☐

  Chambre de prière ☐ Tradi-Praticien ☐
- Date D'arrivée à l'Hôpital : __________________________Heure_____________________

II. ANTECEDENTS

- Formule Obstétricale

G	P	Ev	Dc	Av

- **Age de la Grossesse actuelle** : __________ S.A
- **CPN suivies ? NON** ☐ **OUI** ☐ Nombre_____________________
- Espace inter génésique
  - ☐ < 18 mois
  - ☐ 18-24 mois
  - ☐ > 24 mois
- Nombre des Césariennes Antérieures : ______________
- Indications aux césariennes antérieures :

**1ère**	**2 ème**	**3 ème**	**4 ème**	**5 ème**

- Counseling sur le ***mode d'Accouchement actuel*** avait-été Fait **?**
  - ☐ **NON**
  - ☐ **OUI** **:** césarienne ☐ Accouchement ☐

III. INFOS SUR LE TRAVAIL

- Signes vitaux :

TA	Pouls	FR	T°

- **Examens faits avant l'accouchement** : ☐ **Hb / nombre ________g/l** **Groupe sanguin fait :** OUI ☐
  - ☐ **Echographie**
  - ☐ Plaquettes
  - ☐ **Sérologie VIH**
- BCF : ☐ Normal ☐ Pathologique ☐ Absent
- Parturiente venue en travail : **OUI** ☐ **NON** ☐
- Partogramme ouvert **: OUI** ☐ **NON** ☐
- Epreuve utérine faite **: OUI** ☐ **NON** ☐
- Accouchement vaginal **: OUI** ☐ **NON** ☐
- **Accouchement Par Césarienne** programmée (faite **Avant le travail) : OUI** ☐ **NON** ☐
- **Accouchement Par Césarienne en cours de travail : OUI** ☐ **NON** ☐
  1. césarienne en urgence ☐
  2. césarienne en catastrophe (**Extrême urgence**) ☐
  3. indication___________________________________

IV. DEROULEMENT

- **Date de décision** accouchement et/ ou césarienne _______________Heure__________
- **Heure de Début de la césarienne**____________________**Heure de la fin**__________
- Heure **d'extraction fœtale**____

- Opérateur : ______________________________________Type d'anesthésie___________
- Type d'incision_____________________________ Antibioprophylaxie Faite ☐
- **Césarienne + autres gestes: Non** ☐
  Oui ☐ Hystérectomie ☐ Césarienne-LTB (ligature) ☐
- Constats opératoires :
  - **Adhérences multiples**
  - **Pré rupture**
  - **Rupture utérine**
  - **Apoplexie (Syndrome de Couvelaire)**
  - **Atonie Utérine**
  - **Anomalies placentaires (placentas acreta, percreta, increta)**
  - **Placenta Bas inséré, Prævia**
  - **Lésions intestinales**
  - **Lésions vésicales**
  - **Lésions urétrales**
  - **Autres(Préciser) ___________________________**

V. PRONOSTIC DE LA MERE

- Heure de Réveil : ____________________________Pronostic : Bon ☐ Mauvais ☐
- Transfusion: **Non** ☐
  Oui ☐ **Nombre d'Unités: 1** ☐ **2** ☐ **3** ☐ **4** ☐ **>4** ☐

VI. PRONOSTIC FŒTAL

- **APGAR à la cinquième minute**

O	1-4	5-7	8-10

Poids
_________grs
Sexe :

- Réanimation du Nouveau-Né : **Oui** ☐ **Non** ☐
- Mort macérée ☐ mort/ Fraiche ☐ Mortalité néonatale ☐
- Détresse Respiratoire : **Oui** ☐ **Non** ☐

VII. POST OPERATOIRE

- Réintervention : **Oui** ☐ **Non** ☐
- Decès : **Oui** ☐ **Non** ☐
- **Complications après accouchements** (Basse et césarienne): (CHOCHEZ SI PRESENT)
  - ***Pertes sanguines plus de 1500ml***
  - ***Pertes sanguines entre 1000-1500 ml***
  - ***Hémorragie interne***
  - ***Infection pariétale/Hématome pariétale***
  - ***Eventration, Eviscération***
  - ***Corps étranger***
  - ***Complications anesthésiques***
  - ***Pelvi péritonite***
  - ***OAP***
  - ***Pyélonéphrite***
  - ***Endométrite***
  - ***Occlusion post opératoire, Iléus paralytique***
  - ***Maladie thromboembolique***

VIII. SORTIE

Avant 7J	Au 7è J	Entre 7-14 J	14 à 21 J	Au-delà de 21 j

- contraception proposée : Oui ☐ Non ☐
- Méthode proposée : ____
